# Supplementary material for: Impact of Peripheral Hydrogen Bond on Electronic Properties of the Primary Acceptor Chlorophyll in the Reaction Center of Photosystem I
Source: Int J Mol Sci. 2024 Apr 28;25(9):4815. doi: 10.3390/ijms25094815 (PMC11084960; doi:10.3390/ijms25094815)
Supplement: Supplementary file 1 [file ijms-25-04815-s001.zip › ijms-2954514-supplementary.pdf]

Supplementary Material

Impact of Peripheral Hydrogen Bond on Electronic Properties of the Primary Acceptor Chlorophyll in the Reaction Center of Photosystem I

Lujun Luo [1,†], Antoine P. Martin [2,†], Elijah K. Tandoh [1], Andrei Chistoserdov [3], Lyudmila V. Slipchenko [4], Sergei Savikhin [2,*] and Wu Xu [1,*]

[1] Department of Chemistry, University of Louisiana at Lafayette, Lafayette, LA 70504, USA; luolujun56@163.com (L.L.)
[2] Department of Physics, Purdue University, West Lafayette, IN 47907, USA
[3] Department of Biology, University of Louisiana at Lafayette, Lafayette, LA 70504, USA
[4] Department of Chemistry, Purdue University, West Lafayette, IN 47907, USA
* Correspondence: sergei@purdue.edu (S.S.); wxx6941@louisiana.edu (W.X.); Tel.: +1-337-482-5684 (W.X.); Fax: +1-337-482-5676 (W.X.)
† These authors contributed equally to this work.

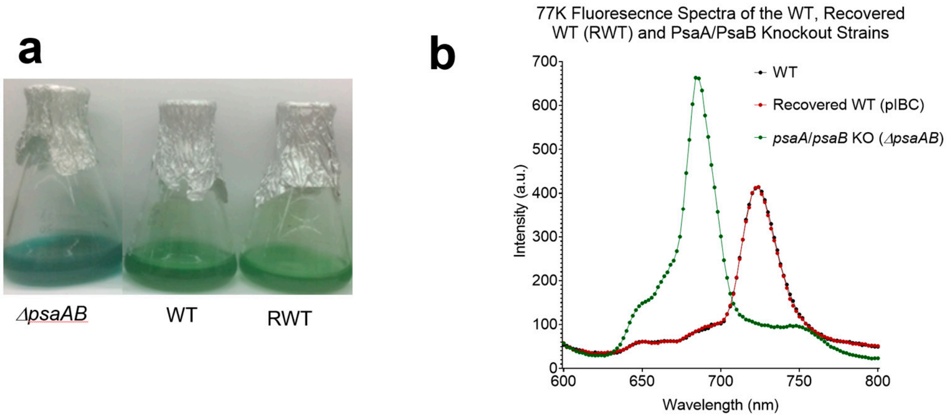

Figure S1. (**a**) The phenotype of Δ*psaAB* is visually dark blue, which is different from the green color of the WT and RWT strains. (**b**) The fluorescence spectrum of the whole mutated cells at 77 K lacked the characteristic PS I emission band at 730 nm, indicating that PS I expression in Δ*psaAB* was highly suppressed or nonexistent. In contrast, the fluorescence spectrum of the pIBC-transformed RWT strain was indistinguishable from the WT.

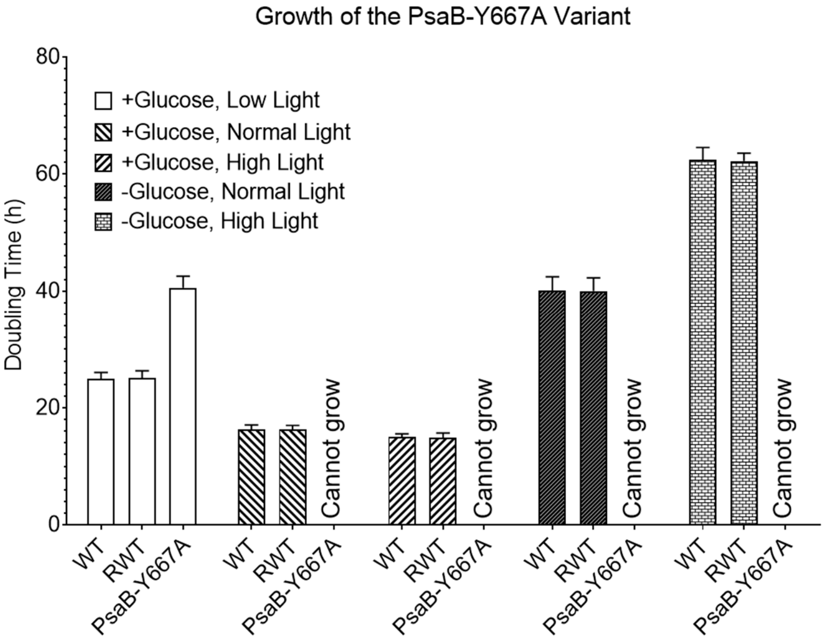

Figure S2. Growth rates of PsaB-Y667A mutants under combinations of normal (40 μmoles m^{-2} s^{-1}) or high light (160 μmoles m^{-2} s^{-1}) in the presence or absence of glucose. Shown also are growth rates of the WT and RWT under similar conditions.

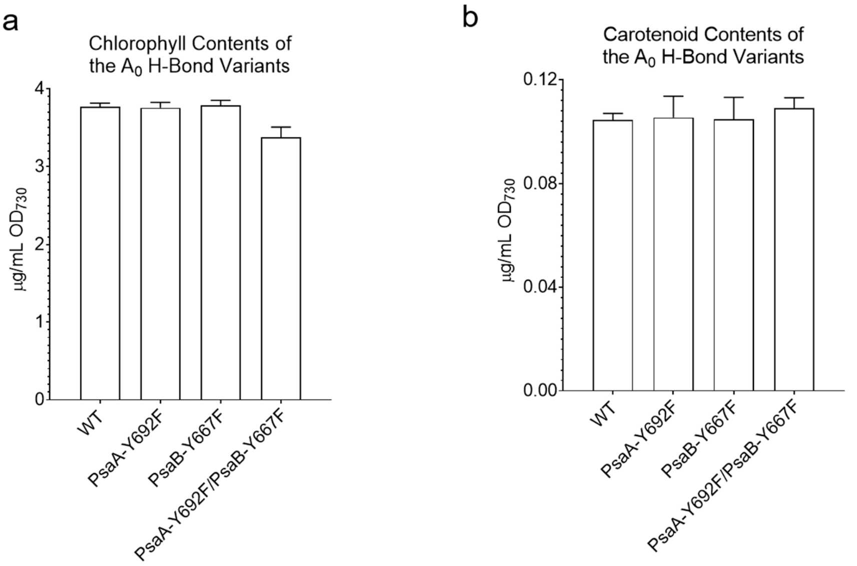

Figure S3. Chlorophyll (**a**) and carotenoid (**b**) content of Y → F mutants is similar to that of the WT.

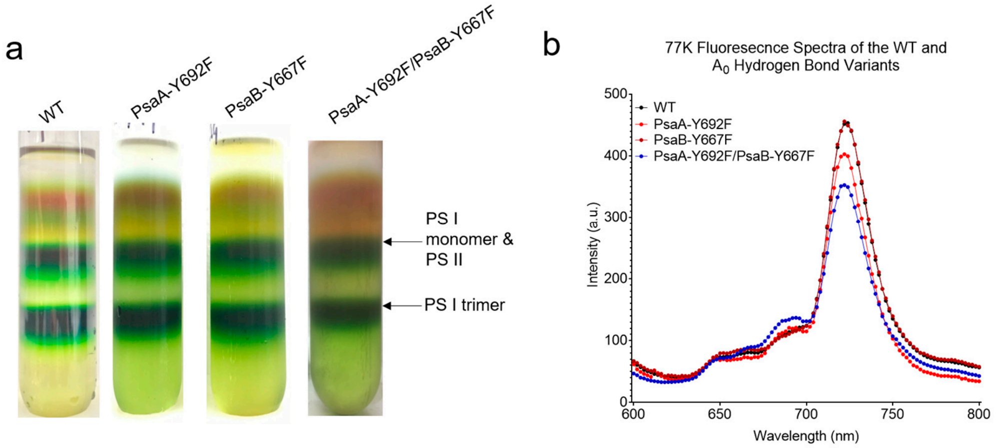

Figure S4. **(a)** Separation of proteins in the WT and Y → F mutants shows strong PS I trimer bands in all species. **(b)** Fluorescence spectra of whole cells at 77K indicate a slightly reduced ratio of PS I and PS II antenna pigments in the A-branch and double mutants. Excitation wavelength is 440 nm.

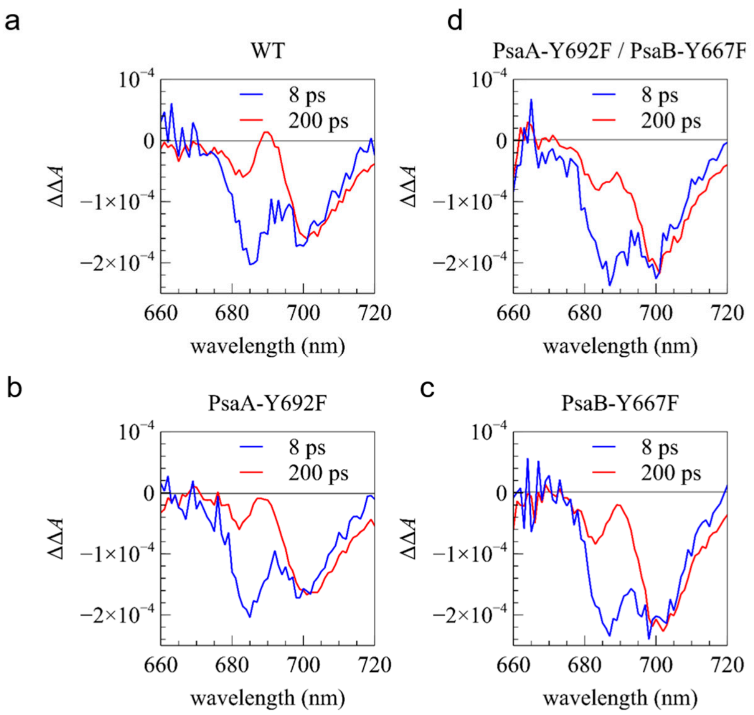

Figure S5. The absorption difference snapshots at 8 ps and 200 ps after exciting PS I at 660 nm. Shown are ΔΔA profiles that are obtained by subtracting profiles measured with closed reaction centers, ΔA_{closed}, from profiles measured with open reaction centers, ΔA_{open}. This procedure extracts signals that correspond to electron transfer processes, since the decay of excitations in antenna has been shown to be similar in the case of open and closed RC, i.e. closed RC acts as a similarly efficient excitation trap as open RC (see Methods in main article). At 200 ps, electron transfer to A_1 is complete forming $P700^+A_0A_1^-$, while at 8 ps it is a mix of $P700^+A_0^-$ A_1 and $P700^+A_0A_1^-$ states, which enables extraction of A_0^- - A_0 spectra as shown in **Figure 7**. ΔΔA profiles show 8 ps and 200 ps spectra measured for the WT (**a**) PsaA-Y692F (**b**), PsaB-Y667F (**c**), and PsaA-Y692F/PsaB-Y667F (**d**).

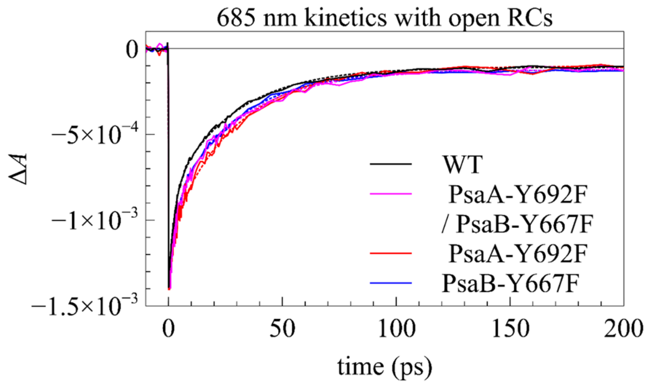

Figure S6. Absorption difference kinetics measured at 685 nm after exciting samples at 660 nm. For all species, the kinetics can be fitted with similar decay components, as listed in Table S1. Slight differences in relative amplitudes of these components may stem from slight differences in absorption spectra of mutants.

Table S1: Exponential decay fit parameters for experiment exciting at 660 nm and probing at 685 nm. Note: A: PsaA-Y692F, B: PsaB-Y667F, and AB: PsaA-Y692F/PsaB-Y667F.

	685 nm kinetic fit parameters	
	lifetime (ps)	relative amplitude (%)
WT	long	8
	28	53
	2.4	39
AB	long	9
	30	58
	2.9	33
A	long	7
	29	70
	2.2	23
B	long	9
	28	60
	2.8	32

Table S2: Electronic excited state calculations of Chl a with Tyr and Phe residues.[a]

	Q_y transition, eV	Q_y transition, cm^{-1}	Shift[b], cm^{-1}
Chl - Tyr	2.3370	18849.6	-6.5
Chl - Phe	2.3352	18835.1	-21.0
Chl	2.3378	18856.1	0.0

[a] Excited state calculations have been performed at the TDDFT wB97x-d/6-31+G* level of theory. Initial structure of the Chl-Tyr complex has been obtained from the PDB ID: 5OY0 (PsaA-Y692), protonated and optimized with harmonic constraints at the wB97x-d/6-31G* level of theory. Geometries of Chl-Phe and Chl have been obtained from the geometry of the Chl-Tyr complex without further reoptimization.

[b] Excitation energy shift with respect to the gas-phase Chl.
